# Investigation of the Urinary Peptidome to Unravel Collagen Degradation in Health and Kidney Disease

**DOI:** 10.1002/pmic.202400279

**Published:** 2024-12-30

**Authors:** Ioanna K. Mina, Luis F. Iglesias‐Martinez, Matthias Ley, Lucas Fillinger, Paul Perco, Justyna Siwy, Harald Mischak, Vera Jankowski

**Affiliations:** ^1^ Mosaiques Diagnostics GmbH Hannover Germany; ^2^ Institute for Molecular Cardiovascular Research University Hospital RWTH Aachen Aachen Germany; ^3^ Systems Biology Ireland, School of Medicine University College Dublin Dublin Republic of Ireland; ^4^ Computational Biology Department Delta4 GmbH Vienna Austria; ^5^ Division of Pediatric Nephrology and Gastroenterology, Department of Pediatrics and Adolescent Medicine, Comprehensive Center for Pediatrics Medical University Vienna Vienna Austria; ^6^ Department of Internal Medicine IV Medical University Innsbruck Innsbruck Austria

**Keywords:** CE‐MS, chronic kidney disease, collagen type I alpha 1 chain, fibrosis, urine

## Abstract

Naturally occurring fragments of collagen type I alpha 1 chain (COL1A1) have been previously associated with chronic kidney disease (CKD), with some fragments showing positive and others negative associations. Using urinary peptidome data from healthy individuals (*n* = 1131) and CKD patients (*n* = 5585) this aspect was investigated in detail. Based on the hypothesis that many collagen peptides are derived not from the full, mature collagen molecule, but from (larger) collagen degradation products, relationships between COL1A1 peptides containing identical sequences were investigated, with the smaller (offspring) peptide being a possible degradation product of the larger (parent) one. The strongest correlations were found for relationships where the parent differed by a maximum of three amino acids from the offspring, indicating an exopeptidase‐regulated stepwise degradation process. Regression analysis indicated that CKD affects this degradation process. A comparison of matched CKD patients and control individuals (*n* = 612 each) showed that peptides at the start of the degradation process were consistently downregulated in CKD, indicating an attenuation of COL1A1 endopeptidase‐mediated degradation. However, as these peptides undergo further degradation, likely mediated by exopeptidases, this downregulation can become less significant or even reverse, leading to an upregulation of later‐stage fragments and potentially explaining the inconsistencies observed in previous studies.

**Abbreviations**
AGEs: advanced glycation end products
CKD: chronic kidney disease
COL1: collagen type I
COL1A1: collagen type I alpha 1 chain
COL1Α2: collagen type I alpha 2 chain
DPP4: dipeptidyl peptidase‐4
ECM: extracellular matrix
eGFR: estimated glomerular filtration rate
MMP: matrix metalloproteinases
PTM: post‐translational modifications
TIMP: tissue inhibitors of metalloproteinases

## 1 Introduction

Fibrosis is a pathological feature of many chronic diseases, including chronic kidney disease (CKD) [1, 2]. It involves the excessive accumulation of extracellular matrix (ECM) components within tissues, ultimately leading to organ dysfunction [1, 2]. Among these components, collagen type I (COL1) is the most abundant protein in the ECM and a major contributor to fibrotic tissue [2, 3]. COL1 is composed of three chains (two alpha 1 [COL1A1] and one alpha 2 [COL1A2]), forming a characteristic triple helical structure [3]. The biosynthesis of COL1 is a complex process of multiple steps, including the hydroxylation of proline and lysine residues, the triple helix formation, the removal of the N‐ and C‐terminal propeptides and the assembly of multiple collagen molecules into fibrils stabilised by intermolecular cross‐linking [4]. The excessive accumulation of COL1 in fibrotic tissues possibly results from an imbalance between COL1 synthesis and degradation [3, 5].

**Summary**

• The current study, after investigating naturally occurring collagen type I alpha 1 chain (COL1A1) degradation fragments in urine, proposes a stepwise degradation process of COL1A1. Initially, the COL1A1 molecule is degraded by endopeptidases, producing larger first fragments, which then undergo further degradation by exopeptidases, resulting in progressively smaller fragments. Notably, the first COL1A1 fragments are consistently downregulated in chronic kidney disease (CKD), indicating an attenuation of endopeptidase‐mediated degradation of COL1A1.

• This study suggests that the accumulation of collagen in kidney fibrosis results not solely from increased collagen expression, but to a substantial degree from impaired collagen degradation. Additionally, the current study explains inconsistencies in earlier studies associating urinary COL1A1 fragments with fibrotic disease, where mostly negative, but also occasionally positive, associations were observed: while the initial degradation of COL1A1 by endopeptidases is downregulated, subsequent further degradation of the COL1A1‐derived peptides by exopeptidases may be increased resulting in some cases in upregulation of smaller peptides.

• As many of these fragments are valuable biomarkers for fibrosis‐related chronic diseases, this study demonstrates the importance of the exact definition of the selected biomarkers, including its C‐ and N‐terminus. Furthermore, understanding the COL1A1 degradation process may provide insights into potential therapeutic targets for treating fibrosis.

Degradation fragments of COL1A1 are the most abundant naturally occurring peptides found in urine [6]. Previous research has linked these peptides to CKD, kidney function and fibrosis [7, 8, 9]. A landmark study from 2010 investigated for the first time differences in urinary peptides between patients with CKD of different aetiologies and controls and described the down‐regulation of COL1A1 peptides as a prominent component associated with CKD [8]. Mavrogeorgis et al. [9] identified 63 COL1A1 fragments that showed a strong positive correlation (rho > 0.3) with estimated glomerular filtration rate (eGFR), while six peptides were negatively correlated (rho < −0.3) with eGFR. Exploring the distribution of urinary peptides in kidney fibrosis, Catanese et al. [7] found two COL1A1 peptides increased in patients with fibrotic disease and three COL1A1 peptides decreased. In a previous study, Magalhães et al. [10] reported three COL1A1 peptides being significant negatively correlated with fibrosis (rho < −0.3). While these findings generally suggest a reduced degradation of COL1A1 in fibrosis, the inconsistencies observed in the relationship between COL1A1 peptides and kidney disease are obvious and unexplained.

Peptidases are enzymes that degrade proteins by hydrolysing peptide bonds and can be classified based on the reactions they catalyse [11]. Exopeptidases require a free N‐terminal amino group, C‐terminal carboxyl group, or both, and cleave peptide bonds within three residues of the polypeptide chain terminus [11]. On the other hand, endopeptidases cleave internal peptide bonds within a polypeptide chain, usually quite distant from the N‐ or C‐terminus [11]. Protease activity is crucial for both, the formation of the mature COL1, and its degradation [3]. Specifically, matrix metalloproteinases (MMPs) including MMP‐1, MMP‐2, MMP‐8, MMP‐9, MMP‐13, MMP‐25 and Cathepsin S are involved in the degradation of COL1 [3]. The degradation of COL1 by MMPs is regulated at multiple levels, including the transcription and post‐translational modifications (PTM) and cellular localisation of the MMPs [3]. Furthermore, MMP activity is also regulated by natural tissue inhibitors of metalloproteinases (TIMPs) [3]. The development of computational tools (such as Proteasix) shed some light on how some of these urinary peptides may be generated [12, 13]. Nevertheless, the specific types of proteases involved in generating urinary COL1A1 peptides remain largely unknown.

A previous study demonstrated that many of the identified COL1A1 urinary peptides are derived from the same regions on the COL1A1 molecule [9]. Additionally, numerous larger peptides contain the entire sequences of smaller ones [9]. This observation led to the hypothesis that smaller COL1A1 peptides are not derived directly from the collagen molecule but may originate from the degradation of larger COL1A1 peptides, and that different proteases may be involved in the generation of the larger and smaller peptides, which may be a plausible explanation for the observed apparent inconsistencies in regulation of collagen peptides in fibrotic diseases. Here, we investigated this hypothesis by exploring ‘parent‐offspring peptide relationships’, where the ‘parent’ (the larger peptide) would be degraded to produce the ‘offspring’ (the smaller peptide). The resulting data indicate that these peptides may indeed be generated in a stepwise manner, primarily regulated by exopeptidases. Furthermore, by investigating the first, larger peptides that are potentially directly derived from collagen through endopeptidase activity (‘first parents’), we found that these peptides were consistently downregulated in CKD, supporting the hypothesis that kidney fibrosis may result at least in part from attenuated collagen degradation.

## 2 Methods

### 2.1 CE‐MS Data

The CE‐MS analysis is described in detail in previous studies [6, 14, 15]. In short, the analysis was performed using a P/ACE MDQ CE system (Beckman Coulter, Fullerton, CA, USA) coupled with a micro‐TOF‐MS (Bruker Daltonics, Bremen, Germany). The raw CE‐MS data were analysed with MosaFinder software, which assigned signals to a list of 21559 peptides, over 5000 of which have known amino acid sequences [16]. To normalise peptide intensities, 29 collagen fragments, which are generally unaffected by disease, were used as internal standards [17].

### 2.2 Dataset Selection

Anonymised urinary peptidome and phenotypic data were retrieved from the Human Urinary Proteome Database [16]. Ethical review and approval were waived for this study (based on the ethics opinion received from the ethics committee of the Hannover Medical School, Germany no. 3116‐2016), due to all data being fully anonymised. For all datasets, eGFR was calculated based on the CKD‐EPI equation [18]. To study normal degradation pathways, datasets from individuals with no evidence of disease and no indication of impaired kidney function (eGFR > 90 mL/min/1.73 m^2^, if available) were selected. To investigate changes in CKD, datasets from patients with impaired kidney function (eGFR < 60 mL/min/1.73 m^2^) were used (Figure S1).

For the analysis of peptide regulation in CKD, urinary peptidome datasets from 1000 CKD patients and 1000 non‐CKD control individuals were randomly extracted from Human Urinary Proteome Database [16]. These datasets were matched for patients’ age, sex and blood pressure (Figure S1).

The analysis focused on the COL1A1 peptides, for which normalised signal intensities were extracted across the selected individuals from the Human Urinary Proteome Database [16]. Intensities of peptides with the same start and stop positions, but with different PTMs (e.g., hydroxylation of proline) were summed up to reflect the total intensity of protein fragments with the same sequence. Only peptides with a frequency >30% in the healthy cohort (*n* = 1131) were shortlisted for all downstream analyses. The frequency threshold of 30% has been found optimal and used in previous peptidome studies to address missing values while maintaining high coverage [19]. The top 10 most abundant peptides with no overlapping sequences in the healthy cohort were selected as ‘anchor’ peptides.

### 2.3 Parent‐Offspring Relationship and Statistical Analysis

Parent‐offspring relationships were determined based on sequence. A relationship was defined if the entire sequence of the offspring peptide was included in the parent peptide sequence.

For each parent‐offspring relationship, Spearman's correlation test was conducted to evaluate the correlation between parent and offspring peptides based on their abundances in the healthy cohort (*n* = 1131). Additionally, a linear model was constructed, with the parent peptide as the independent variable and the offspring peptide as the dependent variable, setting the intercept to zero. This was based on the premise that the absence of the parent peptide would result in the absence of the offspring peptide. The linear models were developed separately for the healthy (*n* = 1131) and CKD (*n* = 5585) cohorts.

To evaluate differences in peptides intensities statistical analysis was performed between matched CKD cases (*n* = 612) and control individuals (*n* = 612) using the non‐parametric Mann–Whitney test. Before the analysis missing abundance values were replaced with 0, as applied in previous studies investigating the peptidome regulation [19].

P‐values were adjusted using the Benjamini–Hochberg (BH) method [20]. All calculations were completed in R (version 4.3.1) using the cor.test, lm, wilcox.test and p.adjust functions from the stats package.

### 2.4 Proteases Analysis

An in‐silico analysis was performed using the peptide‐centric tool Proteasix [12] to predict the endopeptidases potentially involved in generating the COL1A1 peptides. Proteasix reconstructed the cleavage sites at the N‐ and C‐terminal sequences of peptides and predicted the endopeptidases involved in their generation based on established protease/cleavage site (CS) associations [21]. The tool offers two modes: one returns predictions based on experimentally observed protease/CS associations, and the other mode returns predicted protease/CS associations [21]. Both observed and predicted modes of the tool were used, considering only results based on human proteases. Results related to the digestive enzyme pepsinogen A (PGA3) were omitted. PGA3 is activated only in the acidic environment of the stomach, consequently, it is not expected to impact on collagen degradation in kidney disease [22]. Consequently, PGA3 does not appear to be of relevance to this study, any predictions involving this protease likely represent artefacts. The analysis was conducted in 2023 when Proteasix was still operational.

Proteases were assigned as possibly regulating specific parent‐offspring peptide relationships based on two criteria: (i) the offspring peptide was predicted to be generated by the protease, and (ii) the parent peptide contained the complete cleavage site recognised by the protease within its sequence.

## 3 Results

### 3.1 Cohort Characteristics

The healthy cohort used for investigating degradation pathways consisted of 1131 unique samples. The mean eGFR value in this cohort was 103.7 (SD 10.1) mL/min/1.73 m^2^. The healthy cohort had an average age of 41.4 (SD 14.2) years and included 42% males. The CKD cohort, used to investigate degradation in disease, included 5585 samples, all with an eGFR < 60 (mean eGFR 39.8, SD 13.7) mL/min/1.73 m^2^. Average age was 66.7 (SD 13.3) years, with 64% males in the CKD cohort.

Due to the known associations of age, sex and blood pressure with the urinary peptidome [23, 24, 25], a random sample of 1000 individuals with normal kidney function (eGFR > 90 mL/min/1.73 m^2^) and 1000 individuals with impaired kidney function (eGFR <60 mL/min/1.73 m^2^) were extracted. These two cohorts were matched to ensure no significant differences in average age, blood pressure and sex distribution. Consequently, any observed differences in peptide abundance between CKD patients and control individuals are unlikely to be influenced by these factors. Clinical characteristics of the CKD patients (*n* = 612) and non‐CKD control individuals (*n* = 612) used for the peptide regulation analysis are summarised in Table S1. The study population consisted of 377 male CKD patients and 378 male controls. The mean age was 60.8 (SD 11.1) years for CKD patients and 60.4 (SD 10.3) years for control individuals. Mean diastolic blood pressure was 77.9 (SD 11.1) mmHg for cases and 78.2 (SD 8.5) mmHg for controls, while mean systolic blood pressure was 137.0 (SD 17.2) mmHg and 135.2 (SD 14.3) mmHg for cases and controls, respectively. There were no statistically significant differences in sex, age or blood pressure between the two groups. Per design, CKD patients had significantly lower eGFR levels as compared to controls (45.5 (SD 12.3) vs. 99.8 (SD 8.9) mL/min/1.73 m^2^, *p* < 0.001).

### 3.2 Parent‐Offspring Peptide Relationships Indicate an Exopeptidase‐Mediated Degradation of COL1A1 Peptides

**FIGURE 1:**
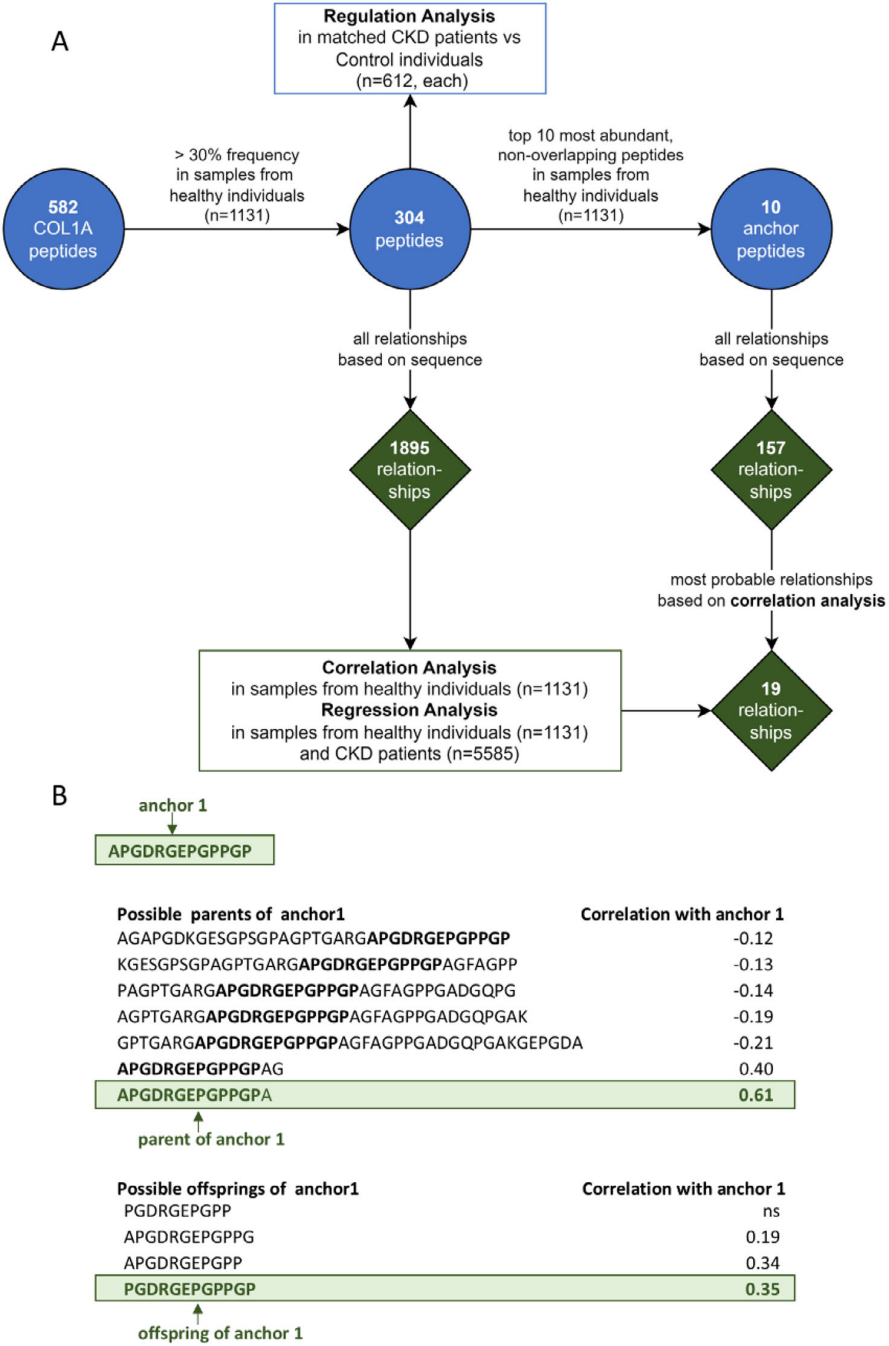
Study design. (A) Out of the 582 identified COL1A1 urinary peptides, 304 peptides passed a 30% frequency threshold in 1131 samples from healthy individuals, being possibly associated with each other in 1895 parent‐offspring relationships based on sequence (where in a relationship the offspring peptide is the degradation product of the parent peptide). For these 304 peptides, the regulation of the peptides in chronic kidney disease (CKD) was investigated using 612 CKD patients and 612 matched control individuals. For the 1895 relationships, the correlations between offspring and parent peptides were investigated in 1131 samples from healthy individuals, and regression analysis between offspring and parent peptides was performed in samples from healthy individuals (*n* = 1131) and CKD patients (*n* = 5585). Because of the large number of peptides and relationships, the top 10 most abundant peptides in samples from healthy individuals (*n* = 1131) with no overlapping sequences were selected and defined as ‘anchor peptides’, possibly involved in 157 relationships based on sequence. For each anchor peptide, one relationship with the anchor as offspring and one relationship with the anchor as parent were shortlisted based on the highest absolute correlation, leading to 19 relationships (one anchor was not involved in any relationships as a parent). (B) Example of shortlisting the relationships for anchor 1. Out of all the possible parents of anchor 1, the parent peptide that had the highest significant correlation with anchor 1 was selected. The same approach was applied to select the offspring of anchor 1. The selected parent and offspring of anchor 1 also show the closest sequence similarity to the anchor among all the other candidates, with only one amino acid difference, a phenomenon that was also common when investigating the other anchors.

The study design is depicted in Figure 1. A total of 788 COL1A1 peptides have been identified in the Human Urinary Proteome Database [9], covering 71% of the full collagen sequence and 95% of the mature collagen sequence. With the exception of two peptides (136–151 and 147–179) that contain N‐propeptide sequences, all identified peptides are derived from the mature COL1A1 sequence, consequently reflecting COL1A1 degradation. COL1A1 is highly modified through proline hydroxylation (most frequent PTM on COL1Α1 peptides), which introduces a mass change of ∼16 Da. This PTM occurs during the COL1A1 biosynthesis and appears to have minimal impact on degradation [9]. Therefore peptides with identical sequences but different position and/or number of PTMs were combined. The 788 COL1Α1 peptides corresponded to 582 unique peptide sequences, with different start and/or stop amino acid position. Of these, 304 peptides were present at frequencies exceeding 30% in the healthy cohort (*n* = 1131 samples). These were considered for the subsequent investigation of COL1A1 degradation processes. Notably, the sequences of many smaller peptides were entirely included in the sequences of larger peptides. This observation suggested a step‐wise degradation process where larger peptides may be degraded to smaller ones. Based on the hypothesis that these smaller peptides are generated from larger ones during the COL1A1 degradation, we focused on such ‘parent‐offspring peptide relationships’. A ‘parent‐offspring relationship’ is defined here by a larger peptide (the parent) that could be degraded into a smaller peptide (the offspring).

Based on peptide sequences, the 304 peptides were involved in 1895 theoretical parent‐offspring peptide relationships, with one peptide being involved in more than one (theoretical) relationships. Investigating this large number of relationships in parallel proved highly complex. To reduce the high number of peptides and the complexity of their possible relations, the top 10 most abundant COL1A1 peptides in the healthy cohort with non‐overlapping sequences were selected as starting points for detailed investigation (Table 1). Due to their high abundance, these 10 peptides (referred in this manuscript as ‘anchor’) are assumed to be of higher stability and representative of the degradation process. Since they originate from distinct, non‐overlapping regions of the COL1Α1 molecule, no parent‐offspring relationships can be observed for the anchor peptides themselves. All anchor peptides were potential offsprings of other peptides, and nine of them (excluding anchor 7 (928–946)) may also function as parents. Anchor 6 (819–843) had the highest number of potential parents (25). Anchor 9 (1008–1041) had the lowest number of potential parents (3) but was involved in the most parent‐offspring relationships as a parent, potentially generating 21 different peptides.

**TABLE 1:** Anchor peptides. Top 10 most abundant non‐overlapping collagen type I alpha 1 chain peptides found in at least 30% of healthy samples (*n* = 1131). Peptide intensities were summed for fragments with the same sequence, but different posttranslational modifications.

Anchor	Start AA	Stop AA	Sequence	Intensity
Anchor 1	798	810	APGDRGEPGPPGP	45,917.63
Anchor 2	432	455	NSGEPGAPGSKGDTGAKGEPGPVG	31,775.09
Anchor 3	543	558	SPGSPGPDGKTGPPGP	29,132.48
Anchor 4	229	249	NGDDGEAGKPGRPGERGPPGP	25,525.88
Anchor 5	1177	1193	VGPPGPPGPPGPPGPPS	23,085.80
Anchor 6	819	843	ADGQPGAKGEPGDAGAKGDAGPPGP	21,917.75
Anchor 7	928	946	PGPAGEKGSPGADGPAGAP	12,175.69
Anchor 8	699	725	ANGAPGNDGAKGDAGAPGAPGSQGAPG	11,464.59
Anchor 9	1008	1041	PPGESGREGAPGAEGSPGRDGSPGAKGDRGETGP	10,963.77
Anchor 10	649	672	AGPPGEAGKPGEQGVPGDLGAPGP	6534.046

Abbreviation: AA, amino acid.

Among the potential parent and offspring peptides identified for each anchor, we aimed to select the peptide that most likely serves as the parent to the anchor (parent of the anchor) and the peptide that is most likely derived from the anchor (offspring of the anchor). Since, in a parent‐offspring relationship, the offspring peptide is the degradation product of the parent peptide, the abundance of the offspring peptide is expected to be influenced by that of the parent, resulting in a strong, statistically significant correlation between the peptide abundances. The peptides with the highest degree of correlation (of abundance) to the anchor peptide were chosen as parent or offspring, respectively. The correlation was assessed in the healthy cohort (*n* = 1131 samples). For each anchor peptide, the relationship with the highest significant absolute correlation was chosen to select the most plausible offspring and parent peptide (Figure 1B). Consequently, a total of 19 relationships were defined (10 parent‐anchor and 9 anchor‐offspring). In these selected relationships, the correlations between parent and offspring peptides were predominantly significantly positive, with the exception of anchor 7 (928–946), which exhibited a significant negative correlation with its parent peptide. Importantly, 17 of the 19 shortlisted relationships involved parent and offspring peptides that shared either the same starting or the same ending amino acid and differed by up to three amino acids at the other end. This pattern indicated that the offspring peptides were likely generated from the parent peptides through exopeptidase activity (Tables 2 and S2).

**TABLE 2:** Parent‐offspring relationships of the 10 anchor peptides.

Offspring	Offspring Start AA	Offspring Stop AA	Parent	Parent Start AA	Parent Stop AA	Rho	Adjusted *p* value
Anchor 1	798	810	Parent of anchor 1	798	811	0.61	2.02E‐112
Offspring of anchor 1	799	810	Anchor 1	798	810	0.35	1.70E‐29
Anchor 2	432	455	Parent of anchor 2	431	455	0.16	1.20E‐07
Offspring of anchor 2	433	455	Anchor 2	432	455	0.43	1.19E‐51
Anchor 3	543	558	Parent of anchor 3	543	560	0.38	2.39E‐38
Offspring of anchor 3	543	556	Anchor 3	543	558	0.23	9.13E‐14
Anchor 4	229	249	Parent of anchor 4	227	249	0.31	1.19E‐25
Offspring of anchor 4	231	249	Anchor 4	229	249	0.65	5.36E‐132
Anchor 5	1177	1193	Parent of anchor 5	1177	1195	0.61	1.33E‐110
Offspring of anchor 5	1178	1193	Anchor 5	1177	1193	0.61	2.18E‐110
Anchor 6	819	843	Parent of anchor 6	818	843	0.41	1.20E‐45
Offspring of anchor 6	820	843	Anchor 6	819	843	0.33	1.47E‐29
Anchor 7	928	946	Parent of anchor 7	916	953	−0.29	3.02E‐21
Anchor 8	699	725	Parent of anchor 8	698	725	0.31	2.94E‐25
Offspring of anchor 8	700	725	Anchor 8	699	725	0.52	1.27E‐76
Anchor 9	1008	1041	Parent of anchor 9	1008	1042	0.51	1.78E‐71
Offspring of anchor 9	1011	1041	Anchor 9	1008	1041	0.63	3.51E‐122
Anchor 10	649	672	Parent of anchor 10	630	674	0.33	5.04E‐28
Offspring of anchor 10	650	672	Anchor 10	649	672	0.66	2.14E‐139

*Note*: Each row represents a potential relationship between an offspring peptide and its parent peptide. The most likely parent‐offspring relationships involving the anchor peptides, determined by sequence and correlation analysis in 1131 healthy samples, are shown. For each anchor peptide, with the exception of anchor 7 which had no identifiable offspring, one relationship with the anchor as ‘offspring’ and one with the anchor as ‘parent’ were shortlisted.

Abbreviation: AA, amino acid.

### 3.3 Possible Alterations of Exopeptidase‐Mediated Degradation of COL1A1 Peptides in CKD

Next, the regulation of anchors and their corresponding parent and offspring peptides, as selected via correlation analysis, was investigated in the context of CKD. For this purpose, peptide abundances were compared between matched cohorts of healthy individuals and patients with CKD, with matching criteria including age, sex, and blood pressure (*n* = 612 each). Of the 10 parent peptides, eight were significantly downregulated in CKD, while one was significantly upregulated. Of the 10 anchor peptides, five were significantly downregulated and one was significantly upregulated. Similarly, among the nine offspring peptides, five were significantly downregulated, and one was significantly upregulated. Overall, a predominance of downregulation in CKD was observed, with this effect being more pronounced at the parent level. In relationships where both, a parent and an offspring peptide, showed significant changes, the regulation trends for the parent and offspring peptides generally aligned. The only exception was the relationship involving anchor 1 (798–810), where the parent peptide was downregulated, while anchor 1 itself was upregulated in CKD (Tables 3 and S2).

**TABLE 3:** Regulation and regression analysis of anchor parent‐offspring relationships in chronic kidney disease (CKD).

Offspring	Fold change	Parent	Fold change	HEALTHY slope	CKD slope	Slope change
Anchor 1	1.12*	Parent of anchor 1	0.67*	2.258*	3.221*	0.962
Offspring of anchor 1	1.11	Anchor 1	1.12*	0.013*	0.016*	0.003
Anchor 2	0.88*	Parent of anchor 2	0.93*	1.171*	1.045*	−0.126
Offspring of anchor 2	0.73*	Anchor 2	0.88*	0.033*	0.050*	0.017
Anchor 3	1.06	Parent of anchor 3	0.81*	7.277*	5.533*	−1.744
Offspring of anchor 3	1.59*	Anchor 3	1.06	0.064*	0.072*	0.008
Anchor 4	0.96	Parent of anchor 4	1.29*	14.844*	9.443*	−5.402
Offspring of anchor 4	0.99	Anchor 4	0.96	0.094*	0.116*	0.022
Anchor 5	0.69*	Parent of anchor 5	0.94	3.740*	1.934*	−1.806
Offspring of anchor 5	0.68*	Anchor 5	0.69*	0.212*	0.324*	0.112
Anchor 6	1.05	Parent of anchor 6	0.95*	5.326*	3.977*	−1.349
Offspring of anchor 6	1.07	Anchor 6	1.05	0.260*	0.262*	0.002
Anchor 7	1.08	Parent of anchor 7	0.57*	11.374*	10.092*	−1.283
Anchor 8	0.72*	Parent of anchor 8	0.54*	6.862*	5.932*	−0.930
Offspring of anchor 8	0.74*	Anchor 8	0.72*	0.466*	0.520*	0.054
Anchor 9	0.63*	Parent of anchor 9	0.43*	8.381*	8.768*	0.387
Offspring of anchor 9	0.85*	Anchor 9	0.63*	0.579*	0.327*	−0.252
Anchor 10	0.92*	Parent of anchor 10	0.53*	2.439*	2.804*	0.366
Offspring of anchor 10	0.85*	Anchor 10	0.92*	0.436*	0.453*	0.017

*Note*: Each row represents a potential relationship between an offspring peptide and its parent peptide, selected from the most likely relationships involving the anchor peptides. The table shows the regulation of both parent and offspring peptides in CKD (CKD vs. Control patients, *n* = 612, each) and regression analysis slope between the parent and offspring peptide in each relationship in healthy (*n* = 1131) and CKD (*n* = 5585) samples. Fold changes are calculated by dividing the average value of peptide intensity in cases and average value of peptide intensity in controls. Slope changes are calculated by subtracting the healthy slope from the CKD slope. Significant fold changes and slopes (*p* < 0.05, Benjamini–Hochberg adjustment) are marked with an asterisk.

To investigate whether the observed changes in peptide abundance may be the result of altered degradation processes in CKD, separate regression analyses were performed for the healthy cohort (*n* = 1131) and the CKD cohort (*n* = 5585). Linear models were developed for each parent‐offspring peptide pair, with the parent peptide as the independent variable and the offspring peptide as the dependent variable. The models demonstrated good predictive performance, with *R* ^2^ values ranging from 0.37 to 0.88 in the healthy cohort and from 0.19 to 0.78 in CKD. The lower *R* ^2^ values in CKD likely are the result of increased variability in the disease state (Table S2). All regression slopes in both healthy and CKD were significantly positive, indicating a positive relationship between parent and offspring peptides. Notably, slopes for relationships between anchors and their parents were consistently larger than those between anchors and their offsprings in both conditions. This finding of low degradation levels of the anchor peptides to produce their offspring explains the high abundance and stability of anchor peptides in urine. In CKD, eight out of nine slopes representing this low anchor degradation were increased, though the difference between healthy and CKD was minimal (≤0.12). Conversely, 7 out of 10 slopes characterising relationships between parent of anchors and anchors decreased in CKD, with reductions ranging from −5.4 to −0.13, indicating a general attenuation of degradation in disease. One of the exceptions was the relationship between anchor 1 and its parent, where the slope increased by 0.96, resulting in the observed downregulation of the parent of anchor 1 and the upregulation of anchor 1 (798‐810) in CKD (Tables 3 and S2).

### 3.4 Degradation of COL1A1 is Downregulated in CKD, Potentially Due to Altered Endopeptidase Activity

The observed more pronounced downregulation of parent peptides in CKD, compared to the anchors and their offsprings, suggested a reduction in the endopeptidase‐mediated degradation of the full COL1A1 molecule. This initial attenuation of degradation may be followed by subsequent degradation steps mediated mainly by exopeptidases, potentially obscuring the impact of the endopeptidase activity. Consequently, some peptides may lose their negative association with CKD or even exhibit a positive association.

**FIGURE 2:**
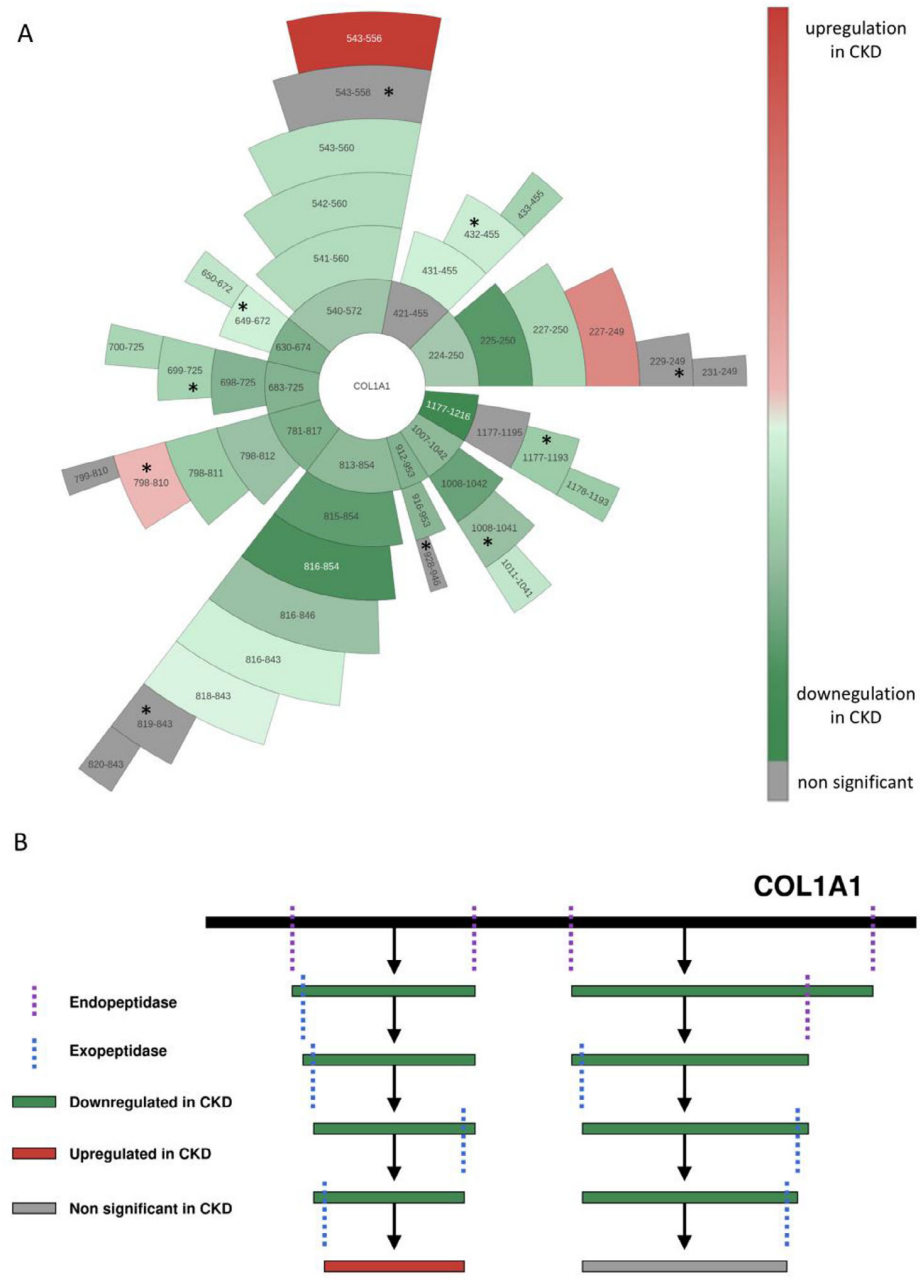
COL1Α1 degradation model. (A) Sunburst plot of COL1A1 parent‐offspring relationships using the OntoloViz tool [58] demonstrating the sequential degradation steps of COL1A1 peptides, where one peptide (symbolised by start and stop amino acid) is degraded into another, ultimately leading to the generation of the anchor peptides (marked with *) and their offsprings. These relationships were determined based on sequence and correlation analysis in samples from healthy individuals (*n* = 1131). The regulation of each peptide in chronic kidney disease (CKD) (using CKD patients vs. Control individuals, *n* = 612, each) is indicated by colour. The initial layer of peptides after COL1A1 represents the first parent peptides, which were consistently downregulated in CKD. (B) Schematic representation of the COL1A1 degradation process, based on the prediction developed here. Initial degradation of the COL1A1 molecule is likely driven by endopeptidases, producing the generation of the first COL1A1 peptides which are consistently downregulated in CKD. These peptides then undergo multiple degradation steps, potentially initiated by endopeptidases but primarily driven by exopeptidases in the later stages. This stepwise process results in smaller peptides that may lose their statistical significance or even exhibit upregulation in CKD, particularly at the last degradation steps.

To test this hypothesis, we examined the regulation of the detected ‘first parent’ peptides, the first identified peptides derived directly from the COL1A1 molecule that initiate the degradation pathways leading to the anchor peptides. We expected that these first parent peptides would consistently show downregulation in CKD. Starting from the anchor peptides, the most probable parent of each anchor based on the highest absolute correlation in the healthy cohort (*n* = 1131) was identified, as described in Section 3.2. This process was repeated iteratively to identify the most probable parent of each parent peptide until the first, most upstream parent for each anchor was determined. The peptide relationships starting from each first parent and leading to each anchor and their offsprings are listed in Table S3 and the entire degradation pathways are depicted in Figure 2A.

On average, each anchor peptide is produced after three degradation steps, with anchor 6 (819–843) requiring the most steps (six consecutive parents) and anchor 10 (649–672) the fewest (one parent). In total, 28 relationships were involved in generating all the anchors from their respective first parents. Nineteen of these relationships involved parent and offspring peptides sharing either the same N‐ or C‐terminal amino acid, differing by up to three amino acids at the other end, indicative of exopeptidase activity. The remaining nine relationships, potentially generated by endopeptidases, often involved the first parent peptides, suggesting a more pronounced impact of the endopeptidase activity at the starting steps of the degradation pathways (Figure 2A and Table S3). Proteasix analysis predicted endopeptidases of the MMP and kallikrein families possibly being responsible for five of these nine relationships (Table S3).

Among the 28 relationships, 25 showed significant positive correlations between parent and offspring peptides, while three, likely generated by endopeptidases based on amino acid differences, showed significant negative correlations. Regression analysis indicated positive slopes for all steps in both healthy and CKD conditions, reflecting the consecutive degradation from parent to offspring peptides. In 6 of the 28 relationships, slopes increased in CKD, while in the remaining 22, slopes decreased, indicating mostly attenuated degradation in CKD (Table S3).

Of the 38 peptides involved in these 28 relationships, two were significantly upregulated, and 30 were significantly downregulated in CKD, showing an overall trend towards downregulation. Importantly, this inconsistency of some peptides being increased in CKD was not observed for the first parents; all nine significantly altered first parents were downregulated in CKD, further supporting the hypothesis of attenuated COL1A1 degradation in CKD (Figure 2A and Table S3).

The first parent peptides either represent fragments of the COL1A1 molecule itself, produced by endopeptidases cleaving within the COL1A1 sequence, or they are fragments of larger COL1A1 peptides not identified by our method. To predict the endopeptidases responsible for generating these first parent peptides, Proteasix analysis was conducted. The analysis indicated that all first parent peptides could potentially be generated by endopeptidase activity, with 5 out of 10 first parents having predicted proteases that cleave both their N‐ and C‐termini. The analysis predominantly predicted members of the MMP as the enzymes responsible for generating these first parent peptides (Table S4).

In contrast, when the same analysis was applied to the final degradation step in the study (the offsprings of the anchor peptides), endopeptidases were predicted to cleave only five out of the nine offspring peptides. Among these, only the offspring of anchor 3 was predicted to be cleaved at both its N‐ and C‐termini by proteases. These findings further support the hypothesis that endopeptidases are likely involved at the beginning of the COL1A1 degradation process but yet undefined exopeptidases are responsible for the generation of the subsequent, smaller COL1A1 degradation products (Table 5).

## 4 Discussion

Fragments from COL1A1 are the most abundant peptides naturally found in urine [6]. While this observation has not been consistently reported in all manuscripts on this topic, the failure of some reports to correctly identify the collagen peptides almost certainly appears to be due to the authors not taking proline hydroxylation into account, consequently not being able of correctly assigning sequence to the respective peptides [26]. Almost all of the identified COL1A1 peptides are fragments of the mature collagen, likely representing COL1A1 degradation process. COL1A1 peptides have been previously described to be associated with fibrosis‐related diseases, including heart failure, CKD and liver diseases, indicating that the degradation of the COL1 molecule is altered in fibrosis [9, 27, 28]. This association is further supported by the fact that some of these peptides have been directly linked to the level of fibrosis in biopsy [7, 10]. However, the relationship between these peptides and fibrotic diseases is not consistent. Although most of the COL1A1 peptides in these studies are negatively associated with fibrotic disease in the kidney, indicating an attenuated COL1 degradation, this pattern is not consistent, some peptides were also described as being significantly upregulated in disease [7, 9, 10]. Also, an increased abundance of peptides from the COL1A1 termini was found associated with death in the context of heart failure, while peptides from the middle part were decreased [29]. Apparently inconsistent associations of COL1A1 peptides has also been reported in the context of liver fibrosis, with most being highly significantly reduced in fibrosis, however, some being increased [27]. The fact that both decreased, but also increased abundance was observed in disease, in fact even in the same samples and both directional changes being highly significant, indicate that this observation cannot be the result of variability, but must be based on some consistent underlying mechanism. Towards a potential explanation of these observed apparent inconsistencies, the current study investigated possible steps of the COL1A1 degradation process leading to peptide generation and how this degradation process is affected in CKD.

Examining the parent‐offspring relationships among COL1A1 peptides, we developed and now propose a model based on sequential degradation processes, where peptides are derived from one another through multiple steps. Correlation analysis between parent and offspring peptides suggested that in most cases each degradation step involves the removal of up to three amino acids from one end of the parent peptide to generate the offspring peptide. This stepwise process continues, with each offspring peptide subsequently becoming a parent peptide itself, undergoing further cleavage of up to three amino acids from one end to produce the next offspring peptide, and continuing in this manner through successive degradation steps. The observed small amino acid differences between parent and offspring peptides in these relationships strongly indicate that these sequential degradation steps may be performed by exopeptidases, enzymes that hydrolyse peptide bonds at the termini of peptide molecules, typically cleaving up to three amino acids at a time [11] (Figure 2B). Regression analysis supported these findings, also indicating that while the degradation process from parent to offspring peptide persists in CKD, its rate may be altered. Specifically, a more pronounced decrease of the exopeptidase‐mediated degradation process was observed in CKD, however, in some cases, exopeptidase activity appeared to increase.

A previous study from Siwy et al. [30] has demonstrated the role of exopeptidase activity in generating collagen peptides in urine. Specifically, in patients with type II diabetes, inhibiting dipeptidyl peptidase‐4 (DPP4), an enzyme that cleaves X‐proline dipeptides from the N‐terminus of polypeptides, using the DPP4 inhibitor linagliptin, led to changes in numerous urinary peptides. Most of these peptides were collagen fragments and analysis of their sequences indicated that they were likely cleavage products resulting from DPP4 activity [30]. Interestingly, DPP4 is implicated in fibrosis across various organs, including the skin, kidneys, liver, heart and lungs, and holds promise as a potential therapeutic target for the treatment of fibrosis [31, 32].

Consistent with previous studies, the abundance of the COL1A1 peptides in CKD showed a general trend towards downregulation, though this trend was not always consistent. Downregulation of peptide abundance was more consistent for the largest peptides, suggesting that the closer a degradation step is to the COL1A1 molecule, the more consistent the downregulation in CKD. To further explore this hypothesis, we traced the larger identified peptides, from which the sequential degradation process begins. These ‘first parent’ peptides likely represent fragments of the COL1A1 molecule itself, produced by endopeptidases that cleave within the COL1A1 sequence. Theoretically they could also be fragments of larger COL1A1 peptides not yet identified, however, we found no evidence for such a scenario. In either case, these first parent peptides, being either direct products of COL1A1 degradation, or closer to this initial degradation than all the other identified peptides, more accurately reflect the initial degradation process of the full COL1A1 molecule. The observed consistent downregulation of these first parent peptides in CKD supports the hypothesis that COL1A1 degradation is attenuated in fibrosis, contributing to the accumulation of COL1A1 in fibrotic tissue (Figure 2B).

Applying Proteasix to predict endopeptidases potentially responsible for generating these first parent peptides resulted in predicting MMPs as the main enzymes involved. MMPs are well‐studied ECM proteases, known for their cleavage of COL1A1 [3]. Interestingly, previous studies suggest an increase of MMPs in CKD. For example, serum MMP‐3 levels were significantly higher in patients with CKD compared to healthy individuals [33]. Similarly, MMP‐2 and MMP‐9 levels were elevated in the plasma of uremic patients [34]. Additionally, MMP‐7 expression was elevated in the kidney tissue of CKD patients [35], with corresponding increases observed in their urinary MMP‐7 levels [36]. Moreover, serum MMP‐10 levels progressively increased in patients with diabetic nephropathy, as the severity of the disease increased [37]. These MMPs may contribute to chronic kidney disease through their inflammatory properties and their role in promoting epithelial‐to‐mesenchymal transition and fibrosis [38]. Since MMPs generally appear to be increased in CKD, the observed attenuation in COL1A1 degradation is unlikely to result from reduced MMP activity. Instead, it may reflect an inability of these MMPs to degrade COL1A1, potentially due to differences in PTMs and collagen crosslinking.

Previous studies have shown that both, the levels and the composition of collagen cross‐linking is altered in fibrosis. For example, elevated expression of lysyl hydroxylase in many types of fibrotic tissue shifts collagen crosslinking toward hydroxyallysine crosslinks at the expense of allysine crosslinks, making collagen more resistant to MMP‐mediated degradation [39, 40]. Moreover, Johnson et al. found a significant correlation between transglutaminase levels and fibrosis in kidney tissues from patients with CKD, likely due to transglutaminase's role in collagen crosslinking, which contributes to collagen resistance against degradation by MMPs [41]. Lysyl oxidase, which is also involved in collagen crosslinking, has been found to correlate positively with the presence and severity of kidney fibrosis in CKD patients, based on both serum and kidney tissue levels [42]. Another factor possibly influencing collagen crosslinking in fibrosis is the accumulation of advanced glycation end products (AGEs). Koska et al. [43] demonstrated that AGEs predict a high risk of CKD in patients with diabetes. Additionally, Makino et al. [44] showed the presence of AGEs in the kidney tissues of diabetic nephropathy patients, which were absent in individuals with no renal disease and in glomerulonephritis patients without diabetes. They also showed that, AGE accumulation, particularly in the mesangium, which contained numerous collagen fibers, increased with the progression of glomerulosclerosis [44]. Other studies have shown that the accumulation of AGEs on collagen reduce its degradation by MMPs, possibly due to structural changes caused by AGE modifications, which disrupt the enzyme–substrate interactions, and AGE‐dependent crosslinks that limit the accessibility of the collagen to proteinases [45, 46].

While increased COL1 production in fibrosis is well‐documented, the role of reduced collagen degradation has received less attention [47, 48, 49]. The findings of this study support the previous hypothesis that collagen accumulation in fibrosis results not solely from increased collagen expression but also from impaired collagen degradation [5]. Failure to resolve excess extracellular matrix, which may be seen as a result of extended ‘wound healing’, appears to be a plausible cause of fibrosis in this context. Therefore, imbalance in collagen homeostasis as underlying the pathology of fibrosis should be carefully considered when developing therapeutic approaches. Therapeutic approaches solely targeting collagen expression may be insufficient if degradation processes are impaired. In such cases, therapies aimed at enhancing collagen degradation might be more effective in managing fibrosis. Further research along these lines appears urgently needed.

Urinary peptidome analysis offers a unique advantage in understanding this aspect. By precisely identifying and measuring COL1 degradation products in urine, this method provides deep insights into the degradation process and its regulation during disease progression. However, it is important to note that the peptide profile observed in urine may not fully reflect the degradation products in tissues. In healthy individuals, 70% of the urinary proteome originates from the kidney and the urinary tract, whereas the remaining 30% represents proteins from the circulation filtered by the glomerulus and not reabsorbed by the renal tubules [50]. Notably, urinary peptidome analysis has enabled developing non‐invasive biomarkers for diagnosis, prognosis and prediction of fibrosis‐related chronic diseases, including CKD and cardiovascular disease [5, 51–54]. Previous urinary peptidome studies have investigated the association between COL1A1 peptides and fibrosis‐related kidney disease [7, 9, 10]. However, these studies reported inconsistencies in peptide regulation, with some peptides showing positive and others negative associations with kidney disease. Our findings provide an explanation for these apparent inconsistencies. Specifically, we observed that downregulation is consistently evident in peptides involved in the earliest degradation step, likely regulated by endopeptidases. However, as degradation progresses, multiple factors, including exopeptidase activity, likely influence the process. This could lead to a loss of significance in downregulation or even upregulation in later degradation steps, explaining the inconsistent peptide regulation observed in previous studies (Figure 2B). This finding has significant implications for proteomic/peptidomic biomarker development. The data demonstrate that the exact definition of the biomarker, based on N‐ and C‐terminus and possibly also present modifications is of the outmost importance. If the detection system relies on just a short stretch of sequence, then in the case presented here, several different peptides will be combined into one signal, even though their regulation is obviously different, potentially resulting in low reproducibility and sometimes puzzling findings. Future studies investigating collagen‐derived biomarkers will benefit from the knowledge of the step‐wise COL1A1 degradation, to guide choosing biomarkers of the highest relevance and consistency, likely peptides closer to the COL1A1 molecule at the first steps of degradation.

The findings should be interpreted within the context of the study's limitations. Firstly, the results are based on statistical assumptions and modelling, not including experimental verification of the COL1A1 degradation process. However, such experimental verification appears extremely challenging, considering the complexity of the process and the multiple proteases potentially being involved. To address this, in vitro experiments could assess the generation of the identified peptides both from the COL1 substrate and from larger synthetic COL1A1 peptides. These experiments, involving combinations of proteases and their specific inhibitors, could validate the stepwise degradation process, demonstrating how smaller peptides are derived from larger ones, and identify the proteases actively participating in collagen degradation. To further explore how collagen degradation is disrupted in CKD, potentially due to increased collagen crosslinking, collagen isolated from kidney tissue of fibrotic animal models and healthy controls could be subjected to enzymatic degradation. Differences in degradation rates would confirm that structural changes in collagen contribute to fibrosis. Therapeutic approaches could then target the prevention of collagen cross‐linking using lysyl hydroxylase, transglutaminase and lysyl oxidase inhibitors and anti‐AGEs therapies [55, 56]. Secondly, the analysis focused on 10 anchor peptides selected for their high frequency and abundance, which may not completely represent the entire spectrum of COL1A1 degradation products. However, the degradation pathways leading to these 10 peptides and their offsprings include 47 peptides, corresponding to 36% of the COL1A1 sequence. This suggests that a representative portion of the COL1Α1 degradation process was captured in this study. Furthermore, this approach can serve as a model for future investigations of the degradation of specific peptides of interest, such as disease‐associated peptides and biomarkers. A third limitation arises from the reliance on in silico tool to predict the enzymes involved in the degradation process. The endopeptidases potentially involved were predicted using Proteasix, based on associations between proteases and (observed or probable) cleavage sites. Although previous studies have validated some of these predictions [13, 57], supporting its reliability, it is important to acknowledge that these predictions would benefit from further experimental validation. We cannot exclude that additional endopeptidases that are not predicted by Proteasix may be involved in the COL1A1 degradation. Furthermore, no tools were available to predict specific exopeptidases involved in the degradation process. Despite these limitations, the model presented, suggesting that endopeptidases primarily regulate the initial steps, while exopeptidases regulate the later steps of COL1A1 degradation, appears the most plausible explanation for the complex findings.

Collectively, investigating the urinary proteome/peptidome enhances our understanding of the COL1A1 degradation process. This could contribute to the development of more effective biomarkers for fibrosis and support identification of potential therapeutic targets to counteract attenuated collagen degradation.

## Conflicts of Interest

H.M. is the founder and co‐owner of Mosaiques Diagnostics GmbH (Hannover, Germany). I.K.M. and J.S. are employed by Mosaiques Diagnostics GmbH. M.L., L.F. and P.P. are employed at Delta4 GmbH (Vienna, Austria).

## Supporting information



Supporting Information

Supporting Information

## Data Availability

The data are not publicly available due to privacy or ethical restrictions. Anonymised data can be made available to investigators after sending a proposal to the corresponding author. Proposals will be reviewed and approved by the investigators, and collaborators on the basis of scientific merit and submitted for approval to the relevant ethics committee.
